# Neighborhood Socioeconomic Deprivation and Gender Disparities in Children with Excessive Body Weight in a Southern European Municipality

**DOI:** 10.3390/children12030321

**Published:** 2025-03-02

**Authors:** Ana C. Lourenço, Helena G. Nogueira, Daniela Rodrigues, Augusta Gama, Aristides M. Machado-Rodrigues, Maria Raquel G. Silva, Cristina Padez

**Affiliations:** 1 CIAS—Reseach Centre for Anthropology and Health, University of Coimbra, 3000-456 Coimbra, Portugal; helenamarquesnogueira@gmail.com (H.G.N.); rodrigues1323@gmail.com (D.R.); maantunes@fc.ul.pt (A.G.); a.machado-rodrigues@fcdef.uc.pt (A.M.M.-R.); raquel@ufp.edu.pt (M.R.G.S.); cpadez@antrop.uc.pt (C.P.); 2 Department of Geography and Tourism, University of Coimbra, 3004-530 Coimbra, Portugal; 3 Department of Life Sciences, University of Coimbra, 3000-456 Coimbra, Portugal; 4 Department of Animal Biology, University of Lisbon, 1749-016 Lisbon, Portugal; 5 Faculty of Sport Sciences and Physical Education, University of Coimbra, 3040-248 Coimbra, Portugal; 6 Faculty of Health Sciences and FP—I3ID, University Fernando Pessoa, 4249-004 Porto, Portugal; 7 CI-IPOP, IPO Porto Research Center—Molecular Oncology and Viral Pathology Group, Portuguese Oncology Institute of Porto, 4200-072 Porto, Portugal; 8 CHRC—Comprehensive Health Research Centre, Nova University of Lisbon, 1150-082 Lisbon, Portugal

**Keywords:** area-level socioeconomic deprivation, excessive weight gain, gender disparities, children, southern Europe

## Abstract

Background/Objectives: Previous research has indicated that gender differences exist in the relationship between neighborhood socioeconomic (SE) deprivation and childhood excessive body weight. However, none of these studies were conducted in a metropolitan area of southern Europe. This study aims to investigate whether the association between neighborhood SE deprivation and childhood excessive body weight in the capital of the Porto Metropolitan Area is influenced by gender. Methods: The sample comprised 832 children (434 girls) aged between 3 and 10 years. Weight and height measurements were taken objectively, and body mass index (BMI) was calculated. The International Obesity Task Force cutoffs were used to identify the children with excessive body weight. Neighborhood SE deprivation was measured using the 2011 Portuguese version of the European Deprivation Index. Logistic regression models were applied for data analysis. Results: Overall, 27.8% of the participating children had excessive body weight. The prevalence of excessive body weight was higher in the neighborhoods characterized by high SE deprivation compared to those with low SE deprivation (34.4% vs. 23.1%). In a multivariable analysis, the girls living in high SE deprivation neighborhoods had a 90% higher risk of excessive body weight compared to the girls in low SE deprivation neighborhoods (OR = 1.90; 95% CI: 1.05–3.44; *p* = 0.035). No significant association was observed between neighborhood SE deprivation and body weight in the boys. Conclusions: The findings indicate that neighborhood SE deprivation substantially increases the risk of excessive body weight, particularly among girls. Therefore, prevention and intervention strategies aimed at addressing excessive body weight gain should specifically target the populations and areas that are at a higher risk.

## 1. Introduction

A growing number of children worldwide are becoming overweight and obese [1,2,3,4]. Between 2015 and 2017, 26.5% of girls and 28.7% of boys aged 7 to 9 years were classified as overweight and obese within the World Health Organization European region. The highest rates of overweight and obesity were recorded in Portugal, Spain, Italy, Greece, and Cyprus, where the prevalence ranged from 32.4% to 43.1% for girls and from 29.0% to 43.0% for boys [5]. Excessive body weight (overweight and obesity) during childhood is a strong predictor of excessive body weight in adulthood and is associated with an increased risk of psychological problems and various non-communicable diseases [6,7,8]. For instance, research from Sweden indicates that girls and boys aged 6 to 17 years with excessive body weight are 1.56 and 2.04 times more likely to experience anxiety or depression symptoms, respectively [9]. A study conducted in Finland demonstrated that excessive body weight after the age of three is linked to over a twofold increase in the risk of developing type 1 diabetes [10]. Furthermore, a study with a sample of 10,003 children aged 5 to 14 years in Northeast India found a significant association between excessive body weight and hypertension (OR = 1.5, 95% CI: 1.1–2.1) [11].

Existing evidence suggests that both genetic and environmental factors play a significant role in the phenomenon of excessive weight gain [12,13,14]. Recent studies have increasingly focused on the impact of neighborhood SE deprivation on childhood excessive body weight [15,16,17,18,19]. Researchers propose that neighborhood SE deprivation may influence children’s body weight statuses by restricting their access to the affordable resources necessary for maintaining healthy physical activity and nutrition, or by increasing their exposure to stressful life events [20,21,22]. Children living in socioeconomically deprived neighborhoods are more likely to experience adverse conditions such as early parental death, financial struggles, persistent discrimination, and exposure to domestic violence, all of which can lead to chronic stress [23,24,25]. The research conducted with both humans and animals demonstrates that chronic stress is linked to excessive body weight through endocrine changes in the body [26,27].

To our knowledge, no studies have investigated the influence of neighborhood SE deprivation on gender differences in childhood excessive body weight within Europe. Understanding how neighborhood socioeconomic deprivation influences the risk of excessive body weight in boys and girls is essential for developing effective interventions to prevent and manage this condition [28]. This issue is particularly relevant in Portugal, as the country recently experienced an economic downturn that resulted in significant cuts to public spending on health and social services [29]. Therefore, the present study investigated whether the association between neighborhood SE deprivation and childhood excessive body weight is influenced by gender, using a sample of children from the city of Porto, the capital of the Porto Metropolitan Area. Porto is situated in northwest region of Portugal, covers an area of 39.74 km^2^, and is subdivided into seven parishes due to the administrative and territorial reform initiated in 2013. In 2019, Porto’s estimated population was approximately 216,606 individuals (97,378 men and 119,228 women), with 28.5% of the residents aged 65 years or older and 7.7% receiving social insertion income benefits [12,30].

## 2. Materials and Methods

### 2.1. Participants and Setting

This cross-sectional study utilizes data from the project “Inequalities in Childhood Obesity: The impact of the socioeconomic crisis in Portugal from 2009 to 2015” (ObesInCrisis). Additional information about this project is available in earlier publications [31,32]. Briefly, data were collected between November 2016 and April 2017 from 118 preschools and primary schools located in the Coimbra, Porto, and Lisbon districts. Participants were selected using proportional stratified random sampling to ensure that the gender distribution of children aged 3 to 10 years was accurately represented across each district. A total of 8472 children (with ages ranging from 3.5 to 11.5 years; 50.8% male) took part in the project, achieving participation rates of 58% in Coimbra, 61% in Porto, and 67% in Lisbon. Ethical approval for the ObesInCrisis project was provided by the Portuguese Commission for Data Protection (authorization number 745/2017) and the Directorate General for Innovation and Curriculum Development (registration number 0565500003). Informed written consent was obtained from the parents or legal guardians of all participating children prior to the data collection phase. Our study concentrates specifically on children aged 3 to 10 years in the municipality of Porto, the capital of the Porto metropolitan area. Initially, 950 children were considered for the analysis, but due to missing home postal codes for 118 children, the final analysis included only 832 participants [12].

### 2.2. Measures

#### 2.2.1. Children Body Weight

Weight was measured to the nearest 0.1 kg using a calibrated scale (Secca, Birmingham, UK), while height was recorded to the nearest 0.1 cm using a portable stadiometer (Leicester Height Measure, Secca, Birmingham, UK). All measurements were conducted by qualified research assistants following a standard protocol, with the children wearing light clothing and no shoes. The body mass index (BMI) of each child was calculated by dividing their body weight in kilograms by the square of their height in meters (kg/m^2^) [33]. The International Obesity Task Force’s gender- and age-specific BMI cutoffs were applied to identify the children with excessive body weight (i.e., those who were overweight or obese) [34].

#### 2.2.2. Neighborhood Socioeconomic Deprivation

In this study, the statistical section served as a proxy for the neighborhood. The municipality of Porto comprises 441 statistical sections. The level of SE deprivation within the neighborhoods was assessed using the 2011 Portuguese version of the European Deprivation Index (EDI-PT 2011). A detailed description of the development of the EDI-PT 2011 can be found in the study by Ribeiro et al. [35]. However, we briefly describe the three key steps involved in the construction of the EDI-PT 2011 below.

Step 1: In this step, a binary indicator for individual deprivation was created using data from the 2011 European Union Statistics on Income and Living Conditions (EU-SILCs) Survey. This measure was based on six fundamental needs that are closely associated with both subjective (the capacity to manage expenses) and objective (income) deprivation in Portugal. The fundamental needs include the ability to have a meal containing fish, meat, or a vegetarian alternative at least once every two days; having the means to handle an unexpected necessary expense easily; maintaining a sufficiently warm home; having access to either a fixed phone or a mobile phone; owning a washing machine; and having a car. Individuals were classified as deprived if they were unable to afford two or more of these six fundamental needs [35,36,37].

Step 2: At this stage, the variables indicating SE deprivation in both the 2011 EU-SILC survey and the 2011 Portuguese Census were identified and organized. Nine variables from the 2011 Portuguese Census matched those in the 2011 EU-SILC survey. These variables include housing tenure status; the availability of flushing toilets in households; the presence of a shower or bath in households; the number of rooms available in households; the occupational class of residents; the highest level of education attained by residents; the employment status of residents; the employment condition of residents; and the nationality of residents. The variables from the 2011 Portuguese Census and the 2011 EU-SILC survey were recoded to ensure comparability. Next, the 2011 Portuguese Census variables were dichotomized. For the variables with multiple categories, logistic regression models were used to determine the optimal dichotomization based on the Wald χ^2^ statistic. Subsequently, the proportions of each variable from the 2011 Portuguese Census were calculated for each statistical section [35,36,37].

Step 3: In this phase, the variables for the EDI-PT 2011 were selected and normalized. A multivariable logistic regression analysis was conducted to identify which of the pre-selected variables from the 2011 Portuguese Census were significantly associated with the individual deprivation indicator. Eight variables demonstrated significant associations with this measure. The regression coefficients from the model were used as the weights for these variables. The EDI-PT 2011 was then calculated as the weighted sum of these eight variables (see Equation (1)), after normalizing them to the national means using the z-score method [35,36,37].

EDI-PT 2011 = 1.191 × %Non-owned households + 1.729 × %Households without flushing toilets + 0.964 × %Households with ≤5 rooms + 0.370 × %Residents in blue-collar jobs + 0.511 × %Residents with ≤6 years of education + 0.620 × %Non-employers + 0.268 × %Unemployed looking for a job + 1.038 × %Foreign residents.(1)

For analysis purposes, the EDI-PT 2011 score was divided into tertiles. Tertile 1 refers to the neighborhoods with the lowest levels of SE deprivation, whereas tertile 3 indicates those with the highest levels of SE deprivation. Each child was assigned to a neighborhood and its corresponding EDI–PT 2011 value based on their home postal code, using a Geographical Information System (QGIS 3.16.6).

#### 2.2.3. Child Sociodemographic Characteristics

The parents completed a questionnaire at home regarding their family characteristics. They were asked to report their own educational attainment as well as that of their spouse or live-in partner. The response options for educational attainment were as follows: (1) illiterate (unable to read and write), (2) 4 years of schooling (first cycle), (3) 6 years (second cycle), (4) 9 years (third cycle), (5) 11 years, (6) professional course, (7) 12 years (secondary education), (8) bachelor’s degree, (9) incomplete bachelor’s degree, (10) master’s degree, and (11) PhD. A family’s educational level was determined by the highest level of education achieved by either parent or by the education level of one parent if the other parent’s information was unavailable. Educational attainment served as a proxy for the family’s socioeconomic position (SEP) and was categorized as follows: low (9 years of schooling or less), middle (10 to 12 years), and high (university qualification) [12]. Moreover, the parents were required to report their child’s gestational age at birth, the child’s number of siblings, and the child’s birth order relative to these siblings. Children born before 37 weeks of gestation were classified as preterm, while those born at 37 weeks or later were classified as term. Birth order was categorized into three groups: only child, firstborn, and not firstborn (which included middle and youngest children) [12].

### 2.3. Statistical Analysis

Descriptive statistics were calculated for the sociodemographic characteristics of children. Comparisons were made between the neighborhoods with low, middle, and high SE deprivation levels using one-way ANOVA and Chi-square tests. Logistic regression models were employed to evaluate the association between neighborhood SE deprivation and excessive body weight. These models were stratified by gender and adjusted for various factors, including a child’s chronological age, birth order, gestational age, and family SEP. To address the possible clustering of children within schools, robust standard errors were calculated using the Huber–White Sandwich estimator. All analyses were carried out using SPSS version 21.0 (SPSS Inc., Chicago, IL, USA). A *p*-value of 0.05 or less was deemed statistically significant.

## 3. Results

The mean age of the children participating in this study was 6.6 years (SD = 1.9), with 52.2% of the sample being girls. Approximately 89% of the children were born at a gestational age of at least 37 weeks, and 27.5% were only children. Excessive body weight was identified in 30.6% of the girls and 24.6% of the boys. Furthermore, around 34% of the children lived in neighborhoods characterized by high levels of SE deprivation. The children from neighborhoods with high SE deprivation were more likely to be classified as having excessive body weight (34.4%) and have parents with low levels of education (39.1%) compared to their counterparts from neighborhoods with low SE deprivation (*p* ≤ 0.05). Detailed characteristics of the sample are provided in Table 1.

Table 2 presents the results from the logistic regression models that examine the relationship between neighborhood SE deprivation and excessive body weight. In the unadjusted models, neighborhood SE deprivation was found to be associated with excessive body weight among boys but not among girls. Specifically, the boys living in neighborhoods with high SE deprivation had 1.77 times higher odds of having excessive body weight (OR = 1.77, 95% CI: 1.01–3.12, *p* = 0.044) compared to the boys in neighborhoods with low SE deprivation. However, after adjusting for factors such as the clustering of children within schools, chronological age, gestational age, birth order, and family SEP, the association became non-significant for the boys but significant for the girls. The girls living in neighborhoods with high SE deprivation had 1.90 times higher odds of having excessive body weight (OR = 1.90, 95% CI: 1.05–3.44, *p* = 0.035).

## 4. Discussion

This study demonstrates that girls living in neighborhoods characterized by high levels of SE deprivation are at an increased risk of having excessive body weight, a trend not observed in boys. Previous research has indicated that neighborhood deprivation impacts the BMI of girls more significantly than it does for boys [38]. For instance, Kowaleski-Jones and Wen [39] analyzed a sample of children aged 2 to 11 years in the United States and found that neighborhood poverty rate was statistically related to body weight status exclusively among girls. Their research indicated that girls living in higher-poverty neighborhoods had a 24% increased risk of having excessive body weight. Similarly, Villa-Caballero et al. [40] reported that girls aged 6 to 13 years attending school in economically disadvantaged neighborhoods were more likely to have excessive body weight, whereas boys showed no similar trend. Another study pointed out that the incidence of excessive body weight among American boys aged 2 to 17 years does increase in alignment with rising neighborhood poverty levels; however, this increase is less significant compared to that observed in their female counterparts within the same age group [41].

The findings presented in this study highlight the differences in parenting choices and practices regarding girls and boys. According to recent statistics from Portugal, only 2.41% of violent crimes against girls are perpetrated by strangers [42]. Despite this low percentage, a substantial number of parents in Portugal continue to perceive girls as more susceptible to criminal victimization, often influenced by beliefs regarding their physical vulnerability. Existing research demonstrates that neighborhoods marked by high levels of deprivation exhibit higher crime rates [43,44]. Consequently, parents residing in such areas may impose stricter restrictions on unsupervised outdoor activities for girls compared to boys. This constriction of outdoor activity after school is associated with increased sedentary behavior and a decrease in engagement in moderate-to-vigorous physical activities [45,46]. Prior studies seem to support the idea that parents often limit their daughters’ outdoor activities to protect them from a potential crime [47,48]. For instance, Miles [49] found that European parents who express feelings of insecurity in their neighborhoods are 2.8 times less likely to encourage their daughters to use the local playgrounds compared to those who feel secure. Considering these findings, it is essential to implement strategies that improve parents’ perceptions of safety from crime, especially in areas with high levels of socioeconomic deprivation. Practical approaches to address the perceived crime rates include demolishing abandoned buildings, improving street lighting, maintaining cleanliness in vacant lots, removing or painting over graffiti, and increasing police presence [50].

We also believe that the observed gender differences may reflect how girls and boys interact with their environment [51]. Boys tend to enjoy activities such as riding bikes or playing football with friends [52], which typically take them beyond their immediate neighborhoods. In contrast, girls are more inclined toward individual and aesthetic activities (e.g., ballroom dancing, artistic roller skating, or rhythmic gymnastics) [53], which typically require specific facilities that are often scarce in deprived neighborhoods [54,55]. Moreover, when such facilities are available, many families may struggle to afford the costs associated with participation [56]. This explanation is supported by the literature showing that girls engage in lower levels of physical activity than boys in neighborhoods with high levels of deprivation [57,58], which may be related to the inadequacy of built environments to meet the specific needs of girls [51]. Therefore, municipalities should increase the availability of free or low-cost after-school sports programs in these neighborhoods [59,60]. However, before implementing such programs, it is crucial to first understand the mechanisms through which neighborhood deprivation contributes to excessive weight gain in girls.

This study highlights the importance of considering neighborhood contextual factors, such as SE deprivation, when implementing strategies aimed at reducing excessive body weight among girls. A notable strength of this research is its reliance on objective measurements of weight and height, which are more reliable than parent-reported data that can often be biased or inaccurate [61]. The assessment of neighborhood SE deprivation was carried out using an ecological index that is both reproducible over time and facilitates comparisons between European countries [62]. Conducted within the context of Portugal, this study adds geographic and cultural diversity to the existing body of literature exploring the impact of neighborhood SE deprivation on excessive weight gain among both girls and boys. Nevertheless, some limitations have been acknowledged. The cross-sectional nature of this study restricts our ability to establish causal relationships. This study analyzed a racially and ethnically homogeneous sample of 832 children, aged 3 to 10 years, living in the city of Porto. To enhance the generalizability of the findings, it would be beneficial to utilize a larger and more diverse sample. Data on the duration of the children’s residence in their neighborhood were not collected; as a result, we were unable to assess the extent of their exposure to SE deprivation. Although BMI is a commonly used metric for assessing excessive body weight, its use may lead to an overestimation of the actual prevalence of excessive weight in our sample. This overestimation arises from the BMI’s inability to differentiate muscle, fat, and bone. Consequently, children with a higher muscle mass or bone density can be incorrectly classified as having excessive body weight [33,63,64]. The non-adjustment for parental BMI is another limitation of this study. We chose not to include parental BMI in our models because we were unable to determine the BMI of most parents due to a lack of reported weight and height data. Finally, the statistical section may not accurately represent a child’s neighborhood [65].

## 5. Conclusions

In conclusion, this study demonstrates that neighborhood SE deprivation may significantly contribute to excessive weight gain during childhood, particularly among girls. The findings show that girls living in neighborhoods characterized by high SE deprivation are 1.90 times more likely to experience excessive weight gain. We hypothesize that this phenomenon may be linked to the features of both the built environment and the social environment in these neighborhoods. Therefore, future research should examine how neighborhood parks, playgrounds, social incivilities, and community cohesion mediate the relationship between neighborhood SE deprivation and excessive body weight in girls. Since girls aged 3 to 10 years are entirely dependent on their parents for participation in recreational activities, future studies should prioritize examining parents’ perceptions of neighborhood environments. Gaining a deeper understanding of the root causes of childhood excessive weight gain is crucial for developing effective prevention strategies in Europe.

## Figures and Tables

**Table 1:** Characteristics of the sample stratified by neighborhood SE deprivation level.

Characteristics	Overall (*n* = 832)	Neighborhood SE Deprivation Level			*p*-Value
		Low (*n* = 277)	Moderate (*n* = 273)	High (*n* = 282)	
Age (years), mean (SD)	6.62 (1.92)	6.44 (1.92)	6.62 (2.01)	6.78 (1.80)	0.106
Gender, *n* (%)					0.136
Male	398 (47.8)	145 (52.3)	129 (47.3)	124 (44.0)	
Female	434 (52.2)	132 (47.7)	144 (52.7)	158 (56.0)	
Gestational age, *n* (%)					0.530
Preterm	87 (11.3)	34 (12.9)	25 (9.8)	28 (11.2)	
Term	682 (88.7)	229 (87.1)	230 (90.2)	223 (88.8)	
Birth order, *n* (%)					0.624
Only child	219 (27.5)	76 (27.8)	79 (30.4)	64 (24.2)	
Firstborn	183 (23.0)	64 (23.4)	56 (21.5)	63 (23.9)	
Non-firstborn	395 (49.6)	133 (48.7)	125 (48.1)	137 (51.9)	
Body weight, *n* (%)					0.007
Normal	601 (72.2)	213 (76.9)	203 (74.4)	185 (65.6)	
Excessive	231 (27.8)	64 (23.1)	70 (25.6)	97 (34.4)	
SEP, *n* (%)					<0.001
Low	223 (29.0)	46 (17.8)	76 (30.2)	101 (39.1)	
Medium	290 (37.7)	99 (38.2)	91 (36.1)	100 (38.8)	
High	256 (33.3)	114 (44.0)	85 (33.7)	57 (22.1)	

SD, standard deviation; SEP, socioeconomic position. The *p*-values refer to the Chi-square test for categorical data and one-way ANOVA test for continuous data.

**Table 2:** Association between neighborhood SE deprivation and excessive body weight stratified by gender.

Neighborhood SE Deprivation Level	Boys			Girls		
	OR	95% CI	*p*-Value	OR	95% CI	*p*-Value
Unadjusted model						
Low	1			1		
Moderate	1.44	0.81–2.54	0.214	0.92	0.54–1.58	0.767
High	1.78	1.01–3.12	0.044	1.63	0.99–2.69	0.055
Adjusted model ^a^						
Low	1			1		
Moderate	1.06	0.53–2.14	0.862	0.88	0.41–1.89	0.733
High	1.32	0.76–2.28	0.303	1.90	1.05–3.44	0.035

SE, socioeconomic; OR, odd ratio; CI, confidence interval; ^a^ models adjusted for the clustering of children within schools, chronological age, gestational age, birth order, and family SEP.

## Data Availability

The data presented in this study are available on request from the corresponding author due to ethical and confidentiality reasons.
